# The Transcriptome Characteristics of Severe Asthma From the Prospect of Co-Expressed Gene Modules

**DOI:** 10.3389/fgene.2021.765400

**Published:** 2021-10-25

**Authors:** Bin Li, Wen-Xuan Sun, Wan-Ying Zhang, Ye Zheng, Lu Qiao, Yue-Ming Hu, Wei-Qiang Li, Di Liu, Bing Leng, Jia-Ren Liu, Xiao-Feng Jiang, Yan Zhang

**Affiliations:** ^1^ Department of Clinical Laboratory, The Fourth Affiliated Hospital of Harbin Medical University, Harbin, China; ^2^ School of Life Science and Technology, Computational Biology Research Center, Harbin Institute of Technology, Harbin, China; ^3^ Heilongjiang Longwei Precision Medical Laboratory Center, Harbin, China

**Keywords:** Phagocytosis-Th2, normal-like, neutrophils, mucin-Th2, Interferon-Th1

## Abstract

**Rationale:** Severe asthma is a heterogeneous disease with multiple molecular mechanisms. Gene expression studies of asthmatic bronchial epithelial cells have provided biological insights and underscored possible pathological mechanisms; however, the molecular basis in severe asthma is still poorly understood.

**Objective:** The objective of this study was to identify the features of asthma and uncover the molecular basis of severe asthma in distinct molecular phenotype.

**Methods:** The k-means clustering and differentially expressed genes (DEGs) were performed in 129 asthma individuals in the Severe Asthma Research Program. The DEG profiles were analyzed by weighted gene co-expression network analysis (WGCNA), and the expression value of each gene module in each individual was annotated by gene set variation analysis (GSVA).

**Results:** Expression analysis defined five stable asthma subtype (AS): 1) Phagocytosis-Th2, 2) Normal-like, 3) Neutrophils, 4) Mucin-Th2, and 5) Interferon-Th1 and 15 co-expressed gene modules. “Phagocytosis-Th2” enriched for receptor-mediated endocytosis, upregulation of Toll-like receptor signal, and myeloid leukocyte activation. “Normal-like” is most similar to normal samples. “Mucin-Th2” preferentially expressed genes involved in O-glycan biosynthesis and unfolded protein response. “Interferon-Th1” displayed upregulation of genes that regulate networks involved in cell cycle, IFN gamma response, and CD8 TCR. The dysregulation of neural signal, REDOX, apoptosis, and O-glycan process were related to the severity of asthma. In non-TH2 subtype (Neutrophils and Interferon-Th1) with severe asthma individuals, the neural signals and IL26-related co-expression module were dysregulated more significantly compared to that in non-severe asthma. These data infer differences in the molecular evolution of asthma subtypes and identify opportunities for therapeutic development.

**Conclusions:** Asthma is a heterogeneous disease. The co-expression analysis provides new insights into the biological mechanisms related to its phenotypes and the severity.

## Introduction

Asthma is a chronic disorder, characterized by airway hyper-responsiveness (AHR) and remodeling with variable degrees of eosinophilic and neutrophilic inflammation resulting in significant morbidity and mortality (Wilson et al., 2006; Kim et al., 2010). It affects about 5% of the population (Global et al., 2017). According to the clinical characteristics, it is mainly divided into the acute and the non-acute asthma, which is further divided into mild, moderate, and severe asthma individual. About 5–10% of the patients do not respond well to standard treatment and have a poor prognosis (Higgins, 2003). The bronchial epithelial cells act as a physical barrier in airway immunity and as central modulators of inflammatory response (Hamilton et al., 2001). Environmental stimuli promote epithelial cell synthesis and secretion by a variety of mediators, such as cytokines, chemokines, reactive oxygen species, lipid, and peptide mediators and eventually involved in recruiting leukocytes, mucus secretion, vascular permeability, bronchoconstriction, and airway hyper-responsiveness (Whitsett, 2018).

Gene expression and genetic variation studies both indicate that asthma is a polygenic and heterogeneous disease with multiple molecular roots (Langfelder and Horvath, 2008; Belsky et al., 2013). Based on the gene profiles in bronchial epithelial cells associated with fractional exhaled nitric oxide (FeNO), Modena et al. identified five phenotypes of asthma. The results showed that a large number of individuals were severe asthma in each subtype (Modena et al., 2014). However, the typical characteristics of phenotype and the features related to severe asthma in phenotype were also unclear. Therefore, revealing these characteristics in each molecular subtype could be valuable for individualized treatment of severe asthma.

In recent years, the WGCNA (Langfelder and Horvath, 2008) (weighted gene co-expression network analysis) is a new system biology approach that can be used to identify co-expression gene sets that largely represent the typical biological characteristics in complex disease. Therefore, in this study, we used the co-expressed gene modules (GMs) that were used to uncover the typical features in subtype and severe asthma.

## Materials and Methods

### Study Population and Data Processing

As part of SARP (Severe Asthma Research Program), bronchial brushing samples and matching demographic data were obtained from 155 participants (129 asthmatics and 26 healthy subjects) from 2009 to 2011. Gene expression of the SARP and external cohorts are available online (GEO database; http://www.ncbi.\.

### K-Means and Limma Analysis

**FIGURE 1:**
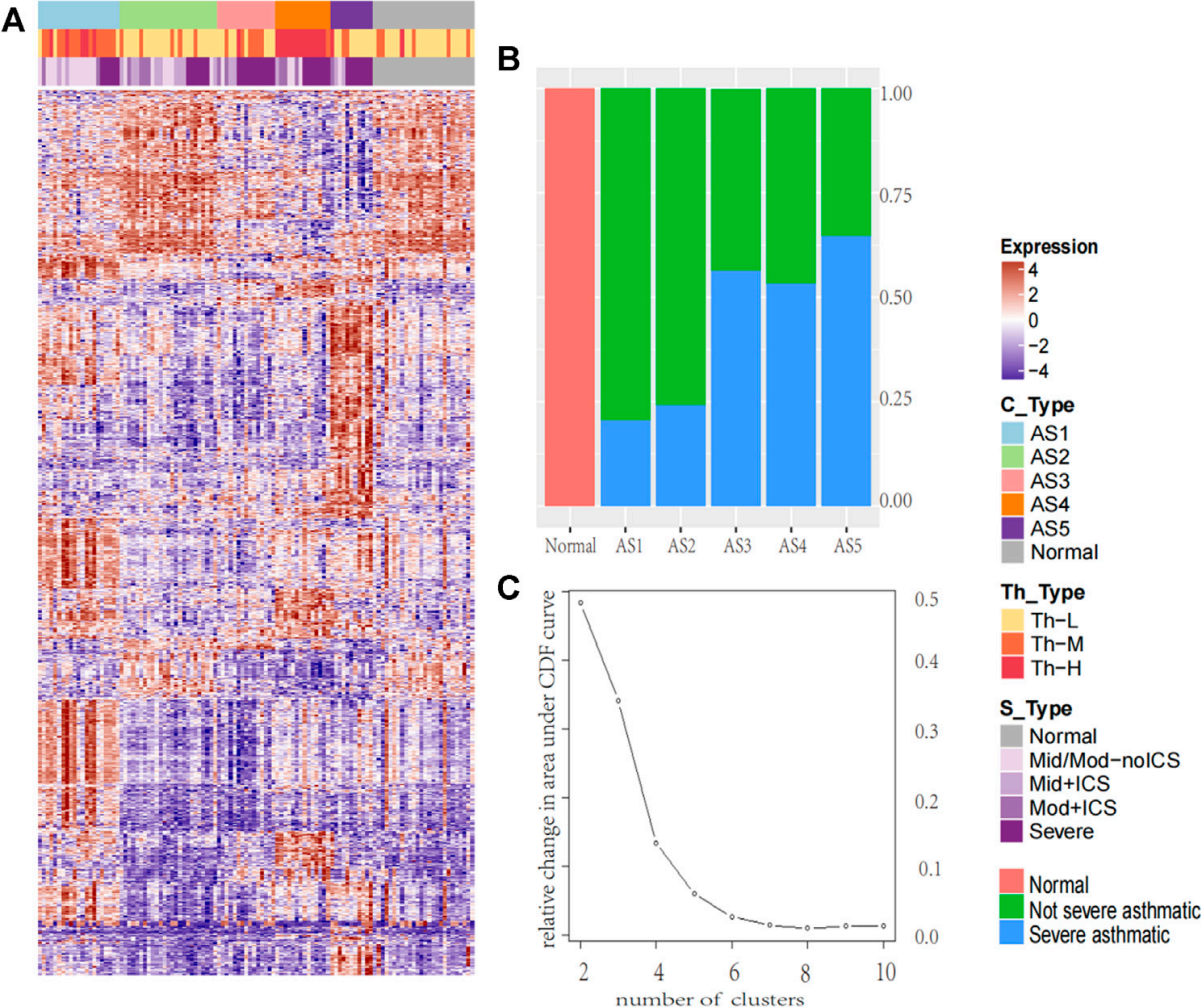
The heatmap of molecular subtype in asthma and the percentage of severe asthma in each cluster. The heatmap of molecular subtype in asthma and the percentage of severe asthma in each cluster **(A)** The k-means identified five stable asthma subtypes (AS1–5), the heatmap was derived by 2,664 DEGs selected in each subtype. To define the Th2 subtype, the unsupervised hierarchical clustering was performed based on the microarray expression levels of periostin (POSTN), channel regulator 1 (CLCA1), and serpin peptidase inhibitor, clade B, member 2 (SERPINB2). We named these subtypes: (1) Th-H, High; (2) Th-M, Moderate; (3) Th-L, Low. According to the severity of the disease, the samples were divided into five groups: Normal for normal group; Mid/Mod-noICS for mild and moderate without ICS treatment; Mid + ICS for mild and ICS treatment group; Mod + ICS for moderate and ICS treatment group; and Severe for severe asthma group **(B)** The percentage of severe asthma in each cluster (red, normal samples, green: non-severe asthma individuals; blue: severe asthma individuals).

According to the transcription of bronchial epithelial cells, the k-means method integrated in the ConsensusClusterPlus (Wilkerson and Hayes, 2010) package was adopted to identify stable subtypes, and the stability was evaluated by iterating for 1,000 times at a sub-sampling rate of 0.95. A total of 4,650 (MAD: median absolute deviation >0.5) genes were used as input. Starting from *k* = 4-5, a significant improvement in clustering stability can be observed, but it has no effect on *k* > 5 (Figure 1C). They were termed by asthma subtype. After the establishment of these ASs, the Bayesian method in Limma package was used to select the differentially expressed genes (DEGs) between each ASs and the normal. The cutoff setting: FC ≥ log (1.5), FDR ≤ 0.05.

To determine the inflammatory Th2 group, K-means on 155 subjects was performed based on microarray expression profiles of three Th2 marker genes (Woodruff et al., 2007) (periostin: POSTN, channel regulator 1: CLCA1, and serpin peptidase inhibitor clade B member 2: SERPINB2). and three main clusters were identified. They were named Th-H (Th2-high), Th-M (Th2-moderate), and Th-L (Th2-low).

### WGCNA Co-expressed Analysis

Using the default parameter setting and the 2,664 DEGs selected in ASs, the WGCNA was performed. This method clusters genes into modules using a topological overlap measure (TOM) (Langfelder et al., 2008). The TOM was a highly robust interconnection measurement method that essentially provided a measure of the connection strength between two adjacent genes and all other genes in a network. Genes were clustered using 1-TOM as the distance measure and GMs were defined as branches of the resulting cluster tree using a dynamic branch-cutting algorithm. Based on the dysregulated direction of each gene in asthma, the genes in each co-expressed module were split into 2 GMs.

### Gene Set Variation Analysis

Gene Set Variation Analysis (GSVA) was performed using the R package “GSVA” (Hänzelmann et al., 2013) (function gsva - arguments: method = “gsva”, mx. diff = TRUE). GSVA implements a non-parametric unsupervised method of gene set enrichment that allowed an assessment of the relative enrichment of a selected pathway across the sample space. The output of GSVA was a gene set by sample matrix of GSVA enrichment scores that were approximately normally distributed. GSVA enrichment scores were generated for each gene set using the normalized gene expression data.

### Pathway Enrichment Analysis

Using the clusterProfiler (Yu et al., 2012) package, the functional enrichment analysis was performed for the up- and downregulated GMs. Significance cutoff was defined as FDR <0.05 for multiple testing.

### Correlation Analysis

The correlation analysis was performed by Pearson. The GSVA score in each gene set represented its overall expression in individual. The C2 (curated gene sets) dataset was downloaded from Molecular Signatures Database (MSigDB), which is a collection of annotated gene sets for pathway analysis (http://software.broadinstitute.org/gsea/msigdb).

## Results

### K-Means and Differentially Expressed Gene Analysis

The k-means was performed, and five stable asthma subtypes were obtained (Figure 1A). These subtypes were named as follows: 1) Phagocytosis-Th2, 2) Normal-like, 3) Neutrophils-Type, 4) Mucin-Th2, and 5) Interferon-Th1 based on the differential expression modules and their related biological clinical characteristics. These five subtypes were associated with specific clinical characteristics (Table 1). “Interleukin-Th2” is the youngest group (mean age = 29) with an elevated FeNO (43 ppb). “Normal-like” has the highest Juniper AQLQ (mean 5, *p* = 4.2E-12). “Neutrophils-Type” has the highest levels of neutrophils in blood (mean, 60, *p* = 0.02) and BAL (mean, 4, *p* = 0.02) and the lowest total cells count in BAL (mean, 2.3, *p* = 3.9E-05). “Mucin-Th2” has the highest FeNO overall (mean, 46, *p* = 2.5E-05) and the greatest reversibility (mean, 21, *p* = 2.5E-05). The “Interferon-Th1” has the highest lymphocytes (mean, 14, *p* = 0.01) in BAL. In addition, three subtypes (“Neutrophils-Type”, “Mucin-Th2”, and “Interferon-Th1”) had more percentage of severe asthma individuals (chi-square, *p* < 0.001) (Figure 1B).

**TABLE 1:** Summary of clinical characteristics of the SARP cohort in AS.

	Normal	AS1	AS2	AS3	AS4	AS5	*p*-value
Inflammatory cells in blood							
Total WBC	5.5 ± 1.3	6.1 ± 1.8	6.4 ± 1.8	7 ± 2.7	6.8 ± 2.9	7 ± 2.9	0.0007
Neutrophils, %	54 ± 6.3	52.8 ± 10	61 ± 8.7	62 ± 15.4	62 ± 12.9	62 ± 13.9	0.02
Basophils, %	1 ± 0.5	0.6 ± 0.5	0.6 ± 0.5	0.2 ± 0.5	1 ± 0.5	1 ± 0.5	0.73
Eosinophils, %	2 ± 1.1	4 ± 2.6	2 ± 1.2	3 ± 4.4	5 ± 2.7	2.5 ± 3.3	0.0009
Lymphocytes, %	33 ± 5.6	34 ± 8.2	31 ± 9.1	26 ± 11.1	29 ± 11.2	26 ± 10.9	0.004
Monocytes, %	8 ± 2.5	8 ± 1.8	7 ± 2	8 ± 4.8	6 ± 1.4	7 ± 2	0.001
Inflammatory cells in BAL							
BAL Total cells	6.1 ± 3.5	8 ± 8.8	7 ± 8.8	2.3 ± 2	4.3 ± 3.6	4.7 ± 8.9	3.9E-05
BAL macrophages,%	86.4 ± 9	91 ± 5.8	90 ± 6.4	85 ± 11.8	89 ± 18.6	81 ± 12.6	0.002
BAL lymphocytes,%	9 ± 7.5	5.8 ± 4.8	7.8 ± 6	9.3 ± 7.2	7 ± 9.5	14 ± 8.3	0.01
BAL eosinophils,%	0.2 ± 1.9	0.4 ± 1.6	0.4 ± 0.7	0.7 ± 1.8	1 ± 2.6	0.3 ± 1.1	0.01
BAL Neutrophils,%	2 ± 4.2	1.3 ± 2.7	1.5 ± 1.8	4 ± 8.2	2 ± 12	2.7 ± 8.7	0.02
Inflammatory cells in sputum							
Total cells, millions	2.1 ± 3.7	1.6 ± 3.8	2 ± 1.4	1.9 ± 5.4	1.4 ± 1.2	1.1 ± 3.6	0.46
Total WBC, millions	1.1 ± 2.2	0.8 ± 3.5	1.3 ± 1	1.3 ± 4.8	1.1 ± 1	0.7 ± 3.6	0.45
Viability of WBCs,%	67 ± 16.6	54 ± 25.9	68 ± 25.7	74 ± 12.2	69 ± 26.9	67 ± 27.8	0.54
Macrophages, %	28 ± 15.1	42 ± 23.8	46 ± 25.9	33 ± 16.1	42 ± 18	42 ± 30.6	0.46
Bronchial epithelial cells,%	3 ± 6	3 ± 9.5	2.5 ± 6.9	2.5 ± 10	5 ± 10.7	1 ± 1.6	0.11
Eosinophils, %	0.9 ± 6.7	0.7 ± 2.9	0.5 ± 10	2.2 ± 15	7.2 ± 11	2.7 ± 1.4	0.40
Lymphocytes, %	1.1 ± 1.4	1.7 ± 3.2	1.4 ± 2.5	1.8 ± 2.2	1 ± 1.5	1.1 ± 1.2	0.82
Pulmonary function and other characteristics							
Baseline FEV1, % predicted	94.5 ± 9	83 ± 16.4	81 ± 24.5	67 ± 25.3	59 ± 19.4	67 ± 18.3	9.9E-08
Baseline FVC, % predicted	96 ± 10.9	93 ± 13.3	85 ± 20.6	81 ± 22.2	69.4 ± 20	89 ± 17.7	0.0009
Maximum FEV1 reversal, %	5.3 ± 3.6	13 ± 16.2	8.7 ± 24	15 ± 38.4	21 ± 27.7	15 ± 13.1	2.5E-05
Juniper AQLQ	7 ± 0.1	4.4 ± 1.2	5 ± 1.3	4.9 ± 1.4	3.8 ± 1.3	3.9 ± 1.1	4.2E-12
Age, years	28 ± 11.9	29 ± 10.7	43 ± 12.2	48 ± 13.7	42 ± 11.1	35 ± 15.5	0.005
Age when first diagnosed	NA	6 ± 10.7	12 ± 14	10 ± 20	13 ± 8.9	9 ± 14.5	0.001
Body mass index	24 ± 5.2	29 ± 5.6	28 ± 6.3	31 ± 6.3	33 ± 10.2	27 ± 6.3	0.052
Number_of_positive_skin_reactions	1.5 ± 3.2	4 ± 3	2 ± 3.2	4 ± 4.1	5 ± 3.6	3 ± 1.9	0.02
FeNO, ppb	21 ± 50.9	43 ± 30.9	17 ± 14.9	43 ± 34.6	46 ± 66.6	22 ± 18.3	2.5E-05

### The Co-expression Features of AS

**FIGURE 2:**
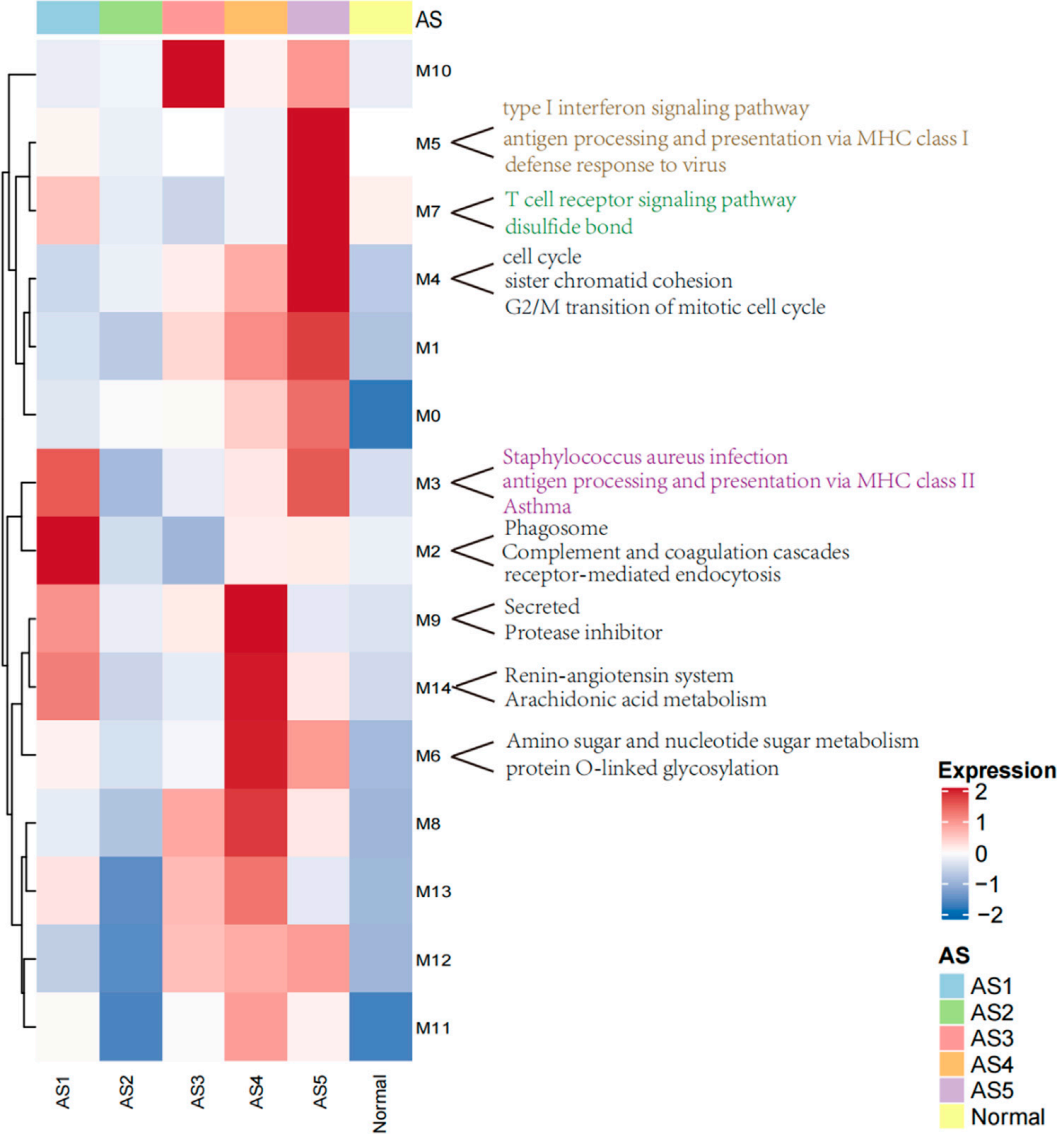
The heatmap of modules with upregulated genes in ASs. The overall expression is represented by red and blue; the red indicates high expressed in cluster, and the blue indicates low expressed in cluster. M0–M14 represents 15 co-expressed modules with upregulated genes.

A total of 2,664 DEGs were detected among these five ASs compared to the normal samples. Using 2,664 DEG as input, a total of 15 coordinately expressed GMs representing distinct biological processes were obtained. In these 15 GMs with only upregulated genes, 10 discriminated these five asthma clusters (Figure 2). In the validation dataset, compared to the normal group, the overall expression level of these modules was consistent with the trend in this study.

### Phagocytosis-Th2 Subtype: AS1

Two core gene programs (GM2 and GM3) characterized “Phagocytosis-Th2”, which included gene networks involved in leukocyte migration (5.3%, FDR = 9.15E-05), osteoclast differentiation (4.5%, FDR = 0.006), receptor-mediated endocytosis (5.7%, FDR = 7.03E-04, COLEC12, MSR1, CD163), and antigen processing and presentation *via* MHC class II (6%, *p* = 1.42E-13, HLA-DMA (Gao et al., 2020), HLA-DRB5, HLA-DMB, HLA-DRB4, HLA-DPB1, HLA-DRA, HLA-DRB3, HLA-DOA, HLA-DQA2 (Lasky-Su et al., 2012), HLA-DRB1, HLA-DPA1). The clinical characteristics showed that the eosinophils (mean: 4, *p* = 0.0009) were abnormally increased in blood. FeNo (mean: 43, *p* = 2.5E-05) levels were higher, while the lymphocyte counts (mean, 5.8, *p* = 0.01) in BAL were lower compared to normal samples. The overall expression of GM2 in this cluster was significantly related to phagocytosis category, which was the reason why it was termed as “Phagocytosis-Th2”.

### Normal-like Subtype: AS2

The Normal-like subtype was the most similar to the normal group, with the mildest clinical symptoms and the fewest differential expressed genes. The top three upregulated genes were PHACTR3 (Itoh et al., 2014), SLCO1B3, and GNMT, which were related to the response of glucocorticoids.

### Neutrophils-type Subtype: AS3

The GM10 was a typical feature of Neutrophils-Type. This module only contained eight upregulated genes, including SLCO1B3, PHACTR3, TPO, and FKBP5 (Binder, 2009), which were also related to the glucocorticoids and severity. The TPO was a marker that related to the severity of asthma (Voraphani et al., 2014).

### Mucin-Th2 Subtype: AS4

Three core gene programs (GM6, GM9, and GM14) characterized “Mucin-Th2”, which included gene networks involved in O-linked glycosylation (2%, *p* = 0.01, i.e., GALNT7, PGM3 (Zhang et al., 2019), and GALNT10), amino acid biosynthetic process (3%, *p* = 0.08, i.e., FOLH1, FOLH1B, and PYCR1), and negative regulation of endopeptidase activity (5.7%, i.e., SERPINA11, SERPINB10, SERPINB2 (Sánchez-Ovando et al., 2020), AHSG, WFIKKN2, and FETUB (Diao et al., 2016)). Individualized functional analysis showed that about 90% of individuals in “Mucin-Th2” significantly enriched Mucin type O-Glycan biosynthesis pathway. This cluster has the highest expression of Th2 marker genes (CLCA1, POSTN, and SERPINB2) and has the highest FeNo overall (median = 46 ppb, *p* = 2.5E-05) and eosinophils in blood, BAL, and sputum (Table 1). Although Mucin-Th2 was the typical Th2, no inflammation and immune-related modules (GM2, GM3, GM5, and GM7) were found overexpressed in this cluster.

### Interferon-Th1 Subtype: AS5

Three core gene programs (GM4, GM5, and GM7) characterized Interferon-Th1, which included gene networks involved in cell division (23%, *p* = 2.39E-29, i.e., ERCC6L, CDCA2, and CDCA3), type I interferon response (15%, *p* = 5.84E-30, i.e., IFITM3, IFITM1, and IFITM2), antigen processing and presentation *via* MHC class I (6%, *p* = 6.53E-09, i.e., HLA-H, HLA-B, HLA-C, HLA-A, HLA-F, B2M, HLA-G, and HLA-E), and T-cell activation (8.9%, *p* = 7.17E-12, i.e., ITK, ZAP70, TNFSF14, CD8B, CD8A, and CD48). The overall expression of GM5 was significantly related to interferon response, which was the reason we termed this type “Interferon-Th1”. This subtype was a typical non-Th2 subtype with normal FeNo (mean:22) and eosinophils in peripheral blood, BAL, and sputum.

### The Characteristics Related to Severity in Asthma

**FIGURE 3:**
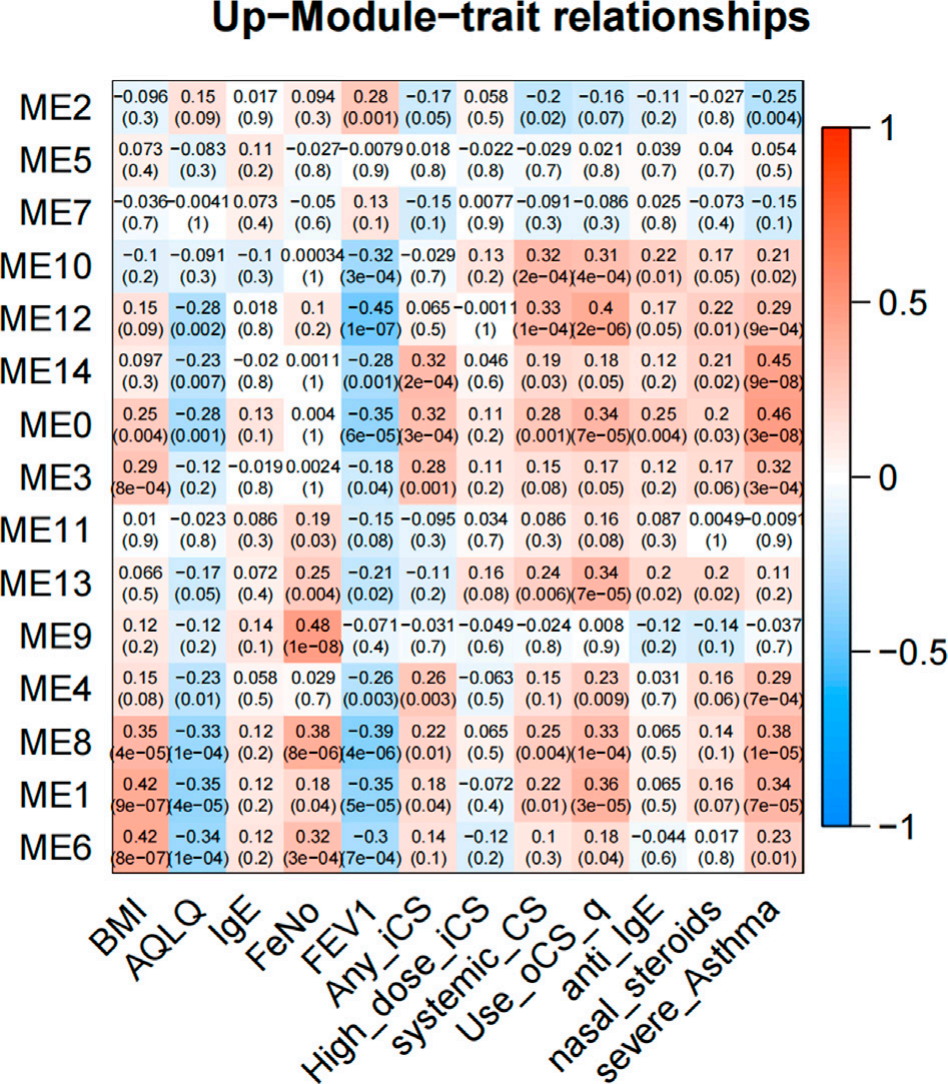
The heatmap of correlation between co-expressed modules with upregulated genes and clinical characteristics. The clinical characteristics include BMI, AQLQ, IgE, fractional exhaled nitric oxide (FeNO), FEV1% predicted, use of inhaled (ICS) and oral corticosteroids (OCS), high dose use of ICS, systemic use CS, anti_IgE treatment, nasal steroids, and the severity of asthma. Positive correlations are red, and negative correlations are blue.

In GMs with upregulated genes, 9 GMs (0, 1, 3, 4, 6, 8, 10, 12, and 14) were positively correlated to the severity and positive association with the use of ICS and OCS. They were also the most negatively correlated with FEV1% predicted and AQLQ (Figure 3). These genes encode proteins related to calcium ion transmembrane transport (6.34%, *p* = 0.003, i.e., P2RY12, LOXHD1, CACNB4, and PKDREJ), apoptotic process (7.26%, *p* = 0.006, i.e., MTFP1, C8ORF4, PTPRH, and LGALS7B), O-glycan processing (4.46%, *p* = 1.33E-06, i.e., GALNT14, MUC1, and MUC2), and amino acid biosynthetic process (3%, *p* = 0.003, i.e., FOLH1, FOLH1B, and PYCR1). In 3 GMs (0, 1, 8), this expression increased with each step of disease severity: healthy control (Normal) < mild-to-moderate asthma not treated with ICS (mild-mod-noICS) < mild-to-moderate asthma treated with ICS (mild-mod-ICS) < severe asthma (severe) (Figure 5).

**FIGURE 4:**
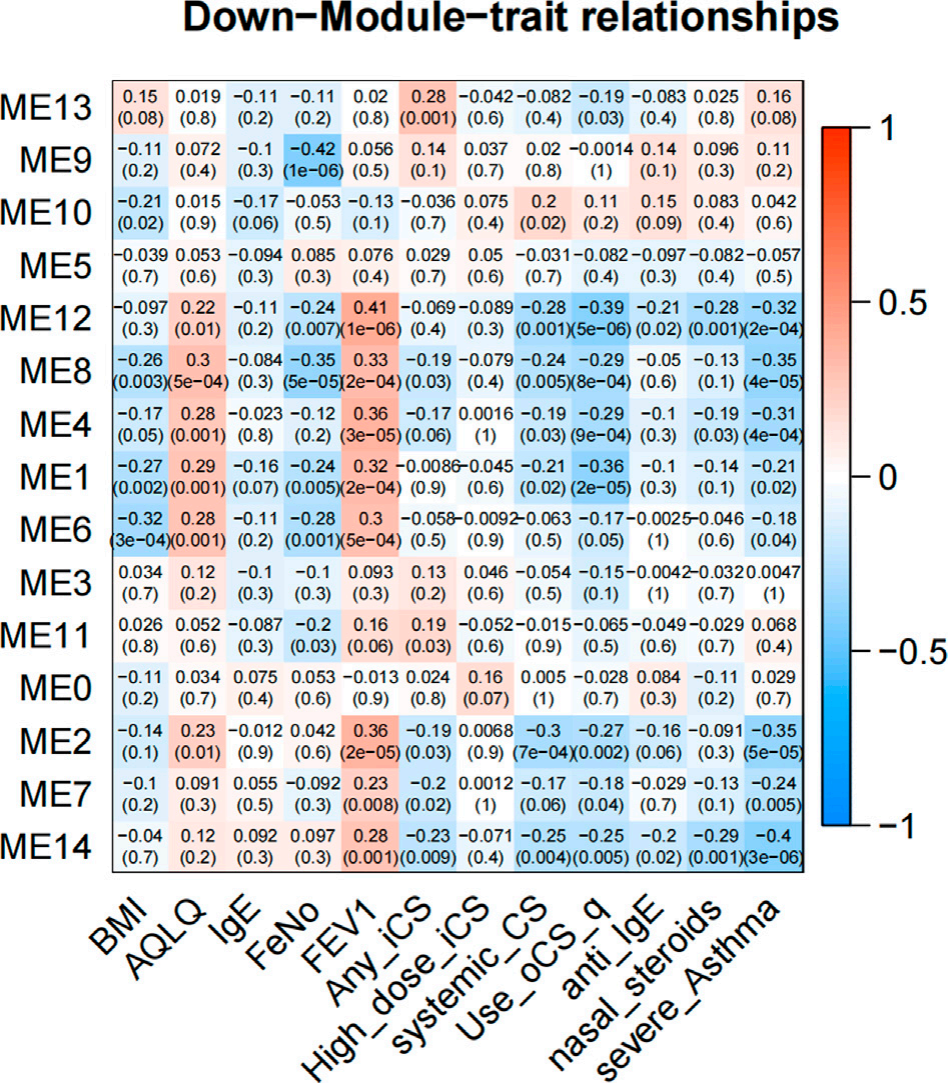
The heatmap of correlation between co-expressed modules with downregulated genes and clinical characteristics. The clinical characteristics include BMI, AQLQ, IgE, fractional exhaled nitric oxide (FeNO), FEV1% predicted, use of inhaled (ICS) and oral corticosteroids (OCS), high dose use of ICS, systemic use CS, anti-IgE treatment, nasal steroids, and the severity of asthma. Positive correlations are red, and negative correlations are blue.

**FIGURE 5:**
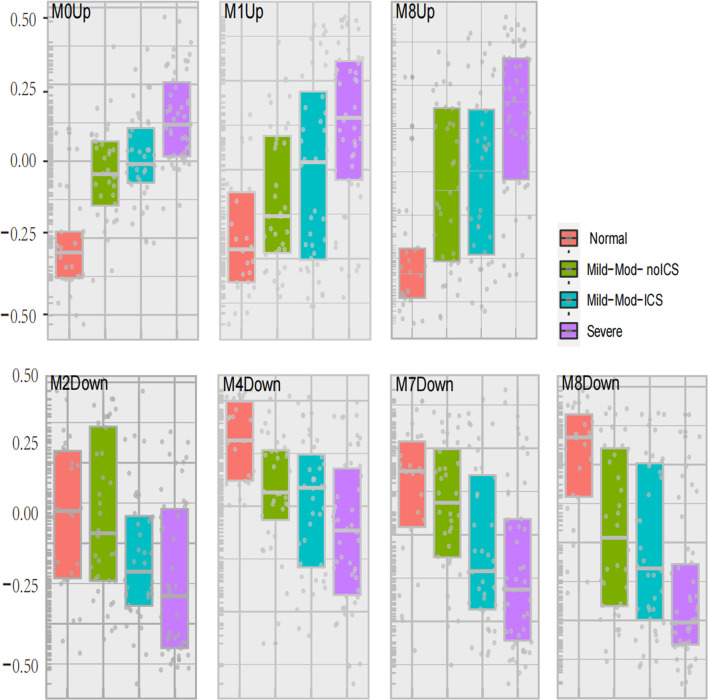
Seven modules expression related to asthma severity. The geometric means were measured according to asthma severity. These classes included healthy controls (Normal), mild-to-moderate asthma not on inhaled corticosteroids (Mild-Mod-noICS), mild-to-moderate asthma on inhaled corticosteroids (Mild-Mod-ICS), and severe asthma (SA).

In GMs with downregulated genes, 8 GMs (1, 2, 4, 6, 7, 8, 12, and 14) were negatively correlated to the severity and negative association with use of ICS and OCS. They were also the most positively correlated with FEV1% predicted and AQLQ (Figure 4). These genes encode proteins related to cell adhesion (5.24%, *p* = 0.002, i.e., CD164, COL16A1, PRKCE, and KIAA1462), innate immune response (10%, *p* = 4.94E-04, i.e., C1QA, MARCO, and SAA1), potassium and sodium ion transmembrane transport (3.7%, *p* = 5.20E-4, i.e., KCNB1, KCNA1, SLC20A2, and SCN11A), lipoprotein metabolic process (5.62%, *p* = 2.05E-5, i.e., LRP1, APOC2, APOC1, LPL, and APOE), cellular oxidant detoxification (5.8%, *p* = 0.01, i.e., GSTM2, GPX3, and CYGB), and neuron signal (10%, *p* = 1.77E-4, i.e., TUBB2B, SPOCK1, and NRCA). In 4 GMs (2, 4, 7, and 8), this expression decreased with each step of disease severity: healthy control (Normal) < mild-to-moderate asthma not treated with ICS (mild-mod-noICS) < mild-to-moderate asthma treated with ICS (mild-mod-ICS) < severe asthma (SA) (Figure 5).

The comparison between mild-mod-ICS and mild-mod-noICS showed that ICS/OCS significantly reduced the expression of 3 GMs (2, 7, and 13) with upregulated genes, while increasing the expression of GM4 with upregulated modules.

### The Severe Characteristics in AS

**FIGURE 6:**
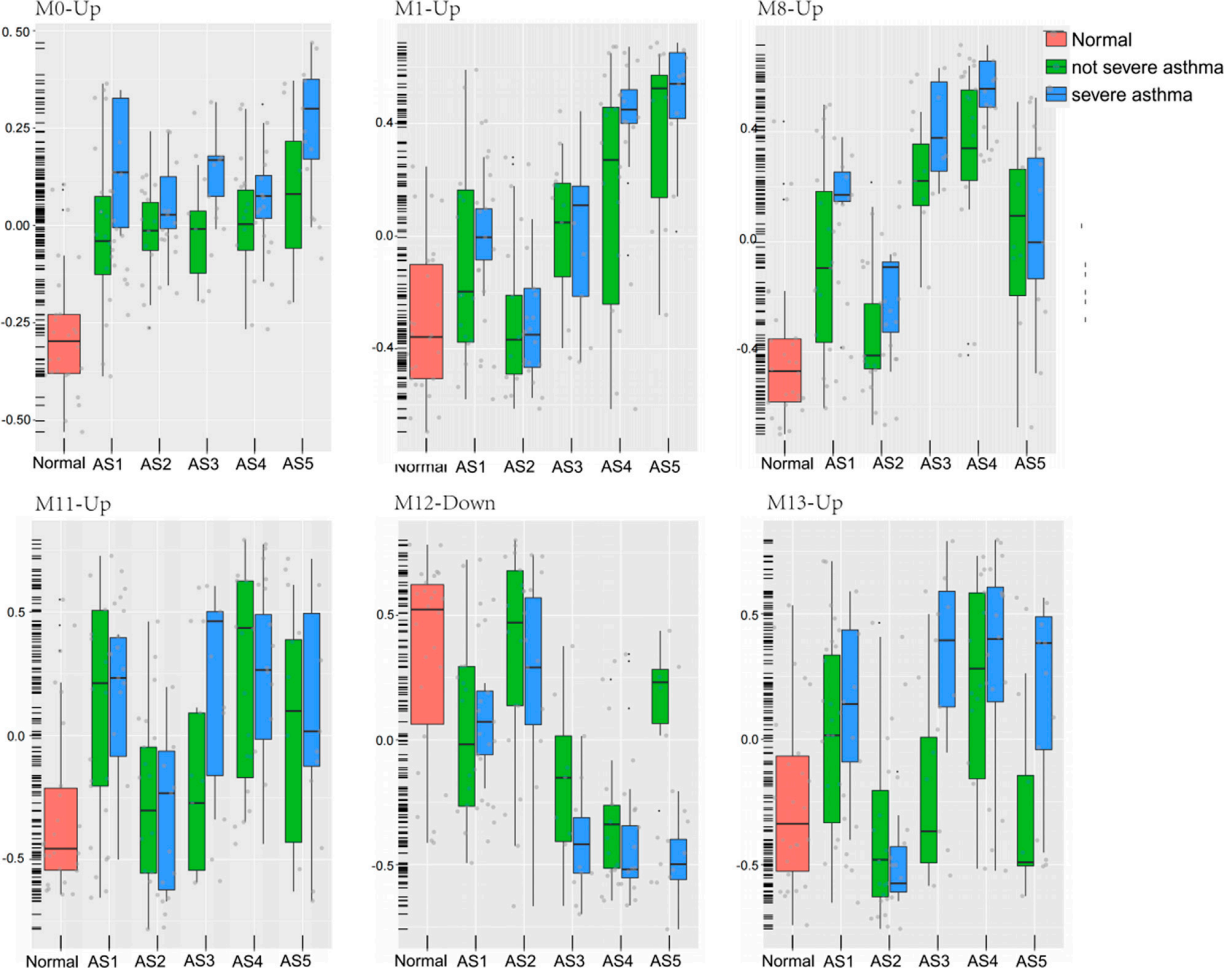
Six modules expression in each phenotype between severe and non-severe groups. The geometric means were measured according to each cluster. These classes included healthy controls (Normal), five asthma phenotypes (AS1–5), non-severe asthma, and severe asthma (SA).

A total of 8 GMs (0-up, 1-up, 8-up, 11-up, 13-up, 8-down, 10-down, 12-down) were significantly different between the severe and non-severe group in specific phenotypes (Table 2), and 6 of them were shown in Figure 6. Compared to that in normal and non-severe samples, the GM0-up related to calcium ion *trans*-membrane transport (6.3%, *p* = 0.004, i.e., P2RY12, LOXHD1 and CACNB4) and negative regulation of endopeptidase activity (4.7%, *p* = 0.04, i.e., SERPINB3 and SERPINB4) was abnormally high expressed in severe asthma across all subtypes.

**TABLE 2:** The differential expression of nine co-expression modules between severe and non-severe individuals in specific subtypes.

AS	Module	meanNonS	meanS	*p*-value
AS1	GM10-Down	−0.159	−0.52	0.02
AS1	GM0-Up	−0.036	0.157	0.01
AS3	GM0-Up	-0.031	0.148	0.008
AS3	GM11-Up	−0.237	0.252	0.016
AS3	GM13-Up	−0.203	0.367	0.005
AS4	GM8-Down	−0.436	−0.551	0.037
AS4	GM1-Up	0.13	0.434	0.018
AS4	GM8-Up	0.298	0.554	0.007
AS5	GM12-Down	0.154	−0.478	8.82E-06
AS5	GM13-Up	−0.314	0.191	0.016

In “Phagocytosis-Th2”, the GM10-down related to nitrogen compound metabolic process (2.06%, *p* = 0.02, i.e., VNN1 and VNN3) was differentially expressed between severe and non-severe groups. In “Mucin-Th2”, the GM1-up related to O-glycan processing (4.7%, *p* = 1.33E-06, i.e., GALNT14, MUC1, and MUC2), GM8-up related to apoptotic process (17%, *p* = 0.01, i.e., LGALS7B, MAL, and SGK1), and GM8-down related to immune response (7.1%, *p* = 0.01, i.e., C3, CXCL6, IL6, and SUSD2) were significantly different between severe and non-severe groups. The GM1-up and GM8-up were much higher expressed while the GM8-down was lower expressed in severe asthma in the “Mucin-Th2” phenotype.

**FIGURE 7:**
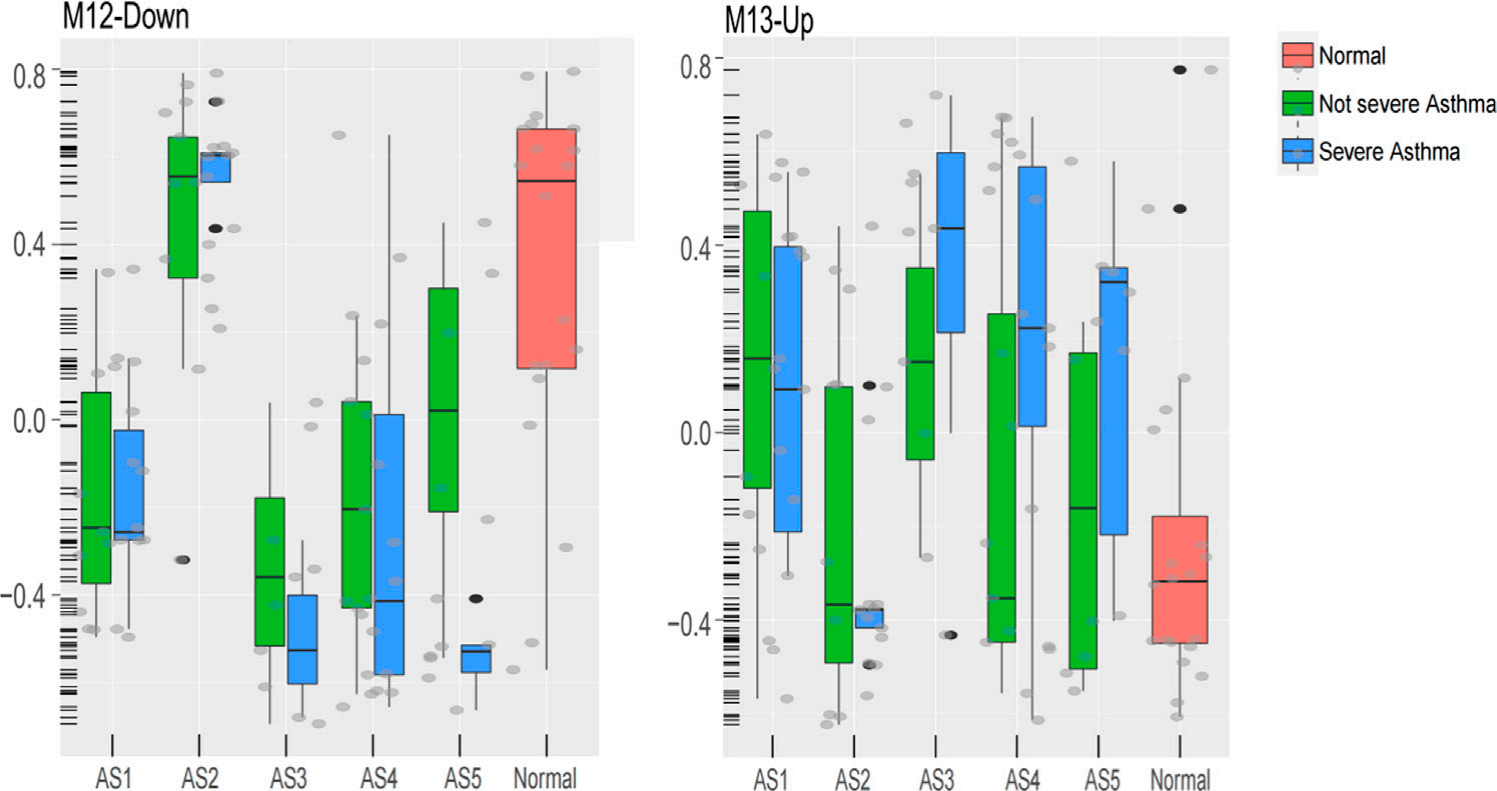
The expression of M13-Up and M12-Down modules in clusters between severe and non-severe groups.

In “Neutrophils-Type” and “Interferon-Th1” (non-Th2) phenotypes, two specific modules (GM13-up and GM12-down) show different expression between severe asthma and non-severe asthma. The GM13-up related to interleukin-26 (IL26, PIK3R5, and LRRC2) was much higher expressed in severe individuals while the GM12-down module related to the nervous system (10%, *p* = 1.77E-04, i.e., TUBB2B, SPOCK1, and ASCL1) was much lower expressed in severe asthma individuals. The similar results were shown in the validation data (Figure 7).

## Discussion

Asthma is a heterogeneous disease with multiple immune and non-immune mechanisms (Ito et al., 2004; McKinley et al., 2008). This study shows that the transcriptome of bronchial epithelial cells was related to ASs and the severity.

Using cluster analysis, five stable ASs were obtained. Multiple clinical features had significant differences among these ASs. These results were similar to that of previous studies (Langfelder and Horvath, 2008). Using the expression of 2,664 DEG profiles as input, the WGCNA co-expression analysis was performed and the module was split based on the dysregulated direction of each gene; 30 GMs were obtained, 15 of which contain only upregulated genes and the other 15 contain downregulated genes (Table 3). These split modules were essential for the description of the typical characteristics in ASs. In each co-expressed module, there was a negative correlation between the two gene sets with up- and downregulated genes, respectively. For example, in GM9 with upregulated genes, multiple Th2-related marker genes (CLCA1, POSTN, etc.) were included, while in GM9 with downregulated genes, the MUC5B (Zhang et al., 2019), SLC28A3, and CSGALNACT1 genes were included and closely related to the reduction of airway defense response (Ridley and Thornton, 2018; Rojas et al., 2019). The MUC5B plays a key role in airway defense. The lack of MUC5B leads to lung inflammation, impaired immune balance, and chronic infection mediated by a variety of bacteria (Roy et al., 2014). These two aspects (up and down features) might be two effective strategies for individualized treatment in asthma.

**TABLE 3:** The number of upregulated and downregulated genes in co-expressed modules.

	Up	Down
Module0	66	86
Module1	185	354
Module2	210	42
Module3	190	85
Module4	194	28
Module5	154	58
Module6	108	56
Module7	118	24
Module8	32	109
Module9	103	25
Module10	8	117
Module11	72	4
Module12	3	50
Module13	43	2
Module14	27	11

Among these 15 modules containing upregulated genes, 4 GMs (2, 3, 5, and 7) were typical immune-related modules and had obvious subtype distribution characteristics. The GM2 and GM3 were typical characteristics of “Phagocytosis-Th2” while the GM5 and GM7 were the typical features of “Interferon-Th1”. The function enrichment analysis showed that the GM2 and GM3 were mainly related to the receptor-mediated endocytosis and antigen presentation *via* MHC-II. The GM5 and GM7 were highly expressed in “Interferon-Th1” and mainly related to type I interferon response and T-cell toxicity. Although the “Mucin-Th2” was a typical Th2 phenotype, the four immune-related modules mentioned above were not significantly increased in this cluster, while the increased glycosylation of O-type glycans, amino sugar and nucleoside sugar metabolism, proteolysis, and unfolded protein reaction were highly expressed in this cluster and positively correlated to the expression of Th2 markers. It suggested that these biological functions could be coordinated with the Th2 signals and related to the physio-pathological mechanisms in “Mucin-Th2”. The increased mucus in airway was a typical clinical feature of Th2 asthma (Dunican et al., 2018; Lambrecht et al., 2019). Studies had shown that the galectin-10 (Galectin-10 and Gal10) crystal structure in airway mucus stimulates the immune system and induces the changes in airway inflammation and mucus secretion (Nyenhuis et al., 2019; Persson et al., 2019). Clearing the crystal structure can effectively relieve airway inflammation and asthma symptoms.

Correlation analysis showed that the apoptotic process and O-glycan processing were positively correlated to asthma severity, while the cell adhesion, innate immune response, potassium and sodium ion transmembrane transport, cellular oxidant detoxification, and neuron signal were negatively correlated to asthma severity. Correcting the imbalance of oxidation and anti-oxidation in the lung may be an important method to relieve asthma symptoms in clinic. GSH is the most important antioxidant in lung tissues (Brigelius-Flohé and Maiorino, 2013). GSH can inhibit a variety of pathogen replication and survival, and increasing the GSH can effectively improve the body’s ability to resist foreign microorganisms (Fitzpatrick et al., 2012). The inhibition of the activity of the CYP450 pathway could destroy the phagocytosis of macrophage and reduces the clearance efficiency of inflammatory stimuli (Bystrom et al., 2013).

In “Phagocytosis-Th2”, the GM10-down was differentially expressed between severe and non-severe groups. In “Mucin-Th2”, the O-glycan processing (GM1-Up), apoptotic process (GM8-Up), and oxidation–reduction process (GM8-down) were significantly different between severe and non-severe groups. The oxygen free radical increase in bronchoalveolar lavage fluid and peripheral blood associated with the severity of disease (Mossberg et al., 2009; Sangiuolo et al., 2015), especially in a typical Th2 phenotype (Huang et al., 2019).

In “Neutrophils-Type” and “Interferon-Th1” (non-Th2) phenotype, interleukin-26 (IL26)-related function was upregulated and related to the severity of asthma. IL-26 is a member of IL-10 cytokine family, is abundant in human airways, and induces the production of pro-inflammatory cytokines (Louhaichi et al., 2020). Stimulation of cultured CD4^+^ T cells with monocyte by recombining IL-26 promoted the generation of RORγ Th17^+^ cells, inducing the production of IL-17A, IL-1β, IL-6, and TNF-α (Louhaichi et al., 2020). Therefore, IL-26 could appear as a novel pro-inflammatory cytokine, produced in airways, and may be a promising target to treat inflammatory asthma.

Although we clustered the transcriptome of bronchial epithelial cells and revealed the typical features in five stable subtypes, the heterogeneity in asthma is much higher than that in subtypes. The characteristics in individuals were more likely a mixture of typical features in multiple subtypes. For asthma, distinguishing subtypes was only a powerful method for uncovering the heterogeneity of complex diseases. Therefore, the individualized analysis based on phenotypes in asthma was a powerful tool for individualized diagnosis and treatment.

## Data Availability

The datasets presented in this study can be found in online repositories. The names of the repository/repositories and accession number(s) can be found in the article.

## Abbreviations

Normal: Healthy controls, AS: asthma subtype, BAl: bronchoalveolar lavage, FeNO: fractional exhaled nitric oxide, ppb: parts-per-billion, Up: the number of upregulated genes in the module, Down: the number of downregulated genes in the module, NonS: the mean value of module in non-severe asthma samples. meanS: the mean value of module in severe asthma samples.
